# Mild traumatic brain injury history is associated with lower brain network resilience in soldiers

**DOI:** 10.1093/braincomms/fcad201

**Published:** 2023-07-27

**Authors:** Jacob R Powell, Joseph B Hopfinger, Kelly S Giovanello, Samuel R Walton, Stephen M DeLellis, Shawn F Kane, Gary E Means, Jason P Mihalik

**Affiliations:** Matthew Gfeller Center, Department of Exercise and Sport Science, University of North Carolina at Chapel Hill, Chapel Hill, NC 27599, USA; Department of Psychology and Neuroscience, University of North Carolina at Chapel Hill, Chapel Hill, NC 27599, USA; Department of Psychology and Neuroscience, University of North Carolina at Chapel Hill, Chapel Hill, NC 27599, USA; Physical Medicine and Rehabilitation, School of Medicine, Virginia Commonwealth University, Richmond, VA 23284, USA; Fort Liberty Research Institute, The Geneva Foundation, Tacoma, WA 98402, USA; Matthew Gfeller Center, Department of Exercise and Sport Science, University of North Carolina at Chapel Hill, Chapel Hill, NC 27599, USA; Department of Family Medicine, University of North Carolina at Chapel Hill, Chapel Hill, NC 27599, USA; United States Army Special Operations Command, Fort Liberty, NC 28303, USA; Matthew Gfeller Center, Department of Exercise and Sport Science, University of North Carolina at Chapel Hill, Chapel Hill, NC 27599, USA

**Keywords:** neurotrauma, neuroimaging, military, networks, connectivity

## Abstract

Special Operations Forces combat soldiers sustain frequent blast and blunt neurotrauma, most often classified as mild traumatic brain injuries. Exposure to repetitive mild traumatic brain injuries is associated with persistent behavioural, cognitive, emotional and neurological symptoms later in life. Identifying neurophysiological changes associated with mild traumatic brain injury exposure, in the absence of present-day symptoms, is necessary for detecting future neurological risk. Advancements in graph theory and functional MRI have offered novel ways to analyse complex whole-brain network connectivity. Our purpose was to determine how mild traumatic brain injury history, lifetime incidence and recency affected whole-brain graph theoretical outcome measures. Healthy male Special Operations Forces combat soldiers (age = 33.2 ± 4.3 years) underwent multimodal neuroimaging at a biomedical research imaging centre using 3T Siemens Prisma or Biograph MRI scanners in this cross-sectional study. Anatomical and functional scans were preprocessed. The blood-oxygen-level-dependent signal was extracted from each functional MRI time series using the Big Brain 300 atlas. Correlations between atlas regions were calculated and Fisher *z*-transformed to generate subject-level correlation matrices. The Brain Connectivity Toolbox was used to obtain functional network measures for global efficiency (the average inverse shortest path length), local efficiency (the average global efficiency of each node and its neighbours), and assortativity coefficient (the correlation coefficient between the degrees of all nodes on two opposite ends of a link). General linear models were fit to compare mild traumatic brain injury lifetime incidence and recency. Nonparametric ANOVAs were used for tests on non-normally distributed data. Soldiers with a history of mild traumatic brain injury had significantly lower assortativity than those who did not self-report mild traumatic brain injury (*t*_148_ = 2.44, *P* = 0.016). The assortativity coefficient was significantly predicted by continuous mild traumatic brain injury lifetime incidence [*F*_1,144_ = 6.51, *P* = 0.012]. No differences were observed between recency groups, and no global or local efficiency differences were observed between mild traumatic brain injury history and lifetime incidence groups. Brain networks with greater assortativity have more resilient, interconnected hubs, while those with lower assortativity indicate widely distributed, vulnerable hubs. Greater lifetime mild traumatic brain injury incidence predicted lower assortativity in our study sample. Less resilient brain networks may represent a lack of physiological recovery in mild traumatic brain injury patients, who otherwise demonstrate clinical recovery, more vulnerability to future brain injury and increased risk for accelerated age-related neurodegenerative changes. Future longitudinal studies should investigate whether decreased brain network resilience may be a predictor for long-term neurological dysfunction.

## Teaser

Graph theory was applied to functional MRI scans collected from 153 Special Operations Forces soldiers to detect brain network impairments associated with mild traumatic brain injury. Lower assortativity coefficients were observed in soldiers with higher brain injury incidence, indicating less resilient brain networks, which may increase the risk for neurodegenerative consequences.

## Graphical Abstract

**Graphical Abstract:**
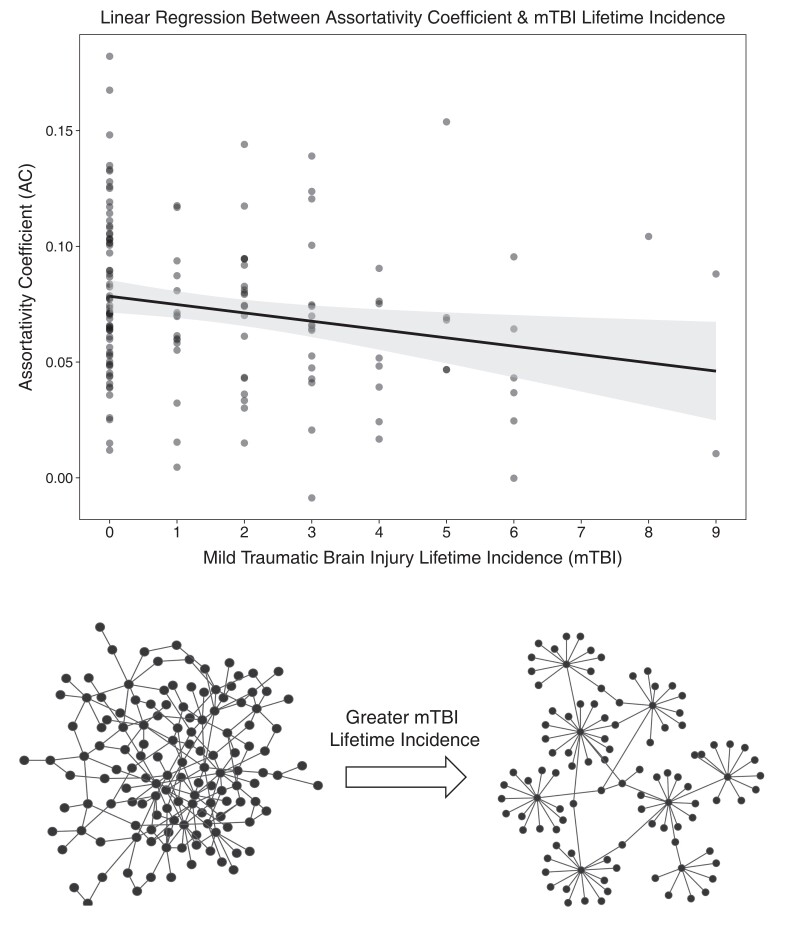

## Introduction

Traumatic brain injuries (TBIs) have been coined the ‘signature injury’ sustained in recent military conflicts.^1^ Between 2000 and 2020, the Traumatic Brain Injury Center of Excellence reported 430 720 TBI diagnoses among the US Armed Forces, of which 354 991 (84.4%) were classified as ‘mild’.^2^ Given the growing reliance on Special Operations Forces (SOF) combat soldiers to intervene in global conflicts, brain injuries may be more frequent in this population, with 25–55% reporting mild TBI (mTBI) history in past studies.^3–5^ Despite the designation ‘mild’ in mTBI, many service members suffer long-term effects. Exposure to repetitive mTBI has been associated with persistent emotional, cognitive, behavioural and neurological symptoms.^6–8^ Growing literature suggests a link between even a single mTBI and an increased risk for neurostructural changes and accelerated age-related neurodegeneration.^9^ Without objective, physiological criteria, determining the effects of mTBI becomes complicated by the heterogenous symptom presentation and reliance on patients’ self-reporting symptom burden.^10,11^ Furthermore, neurophysiological recovery may require more time than clinical recovery.^12,13^ This incongruence between symptom expression and neurobiological healing reinforces the need for objective assessments to determine the lasting subclinical changes linking acute mTBI exposure to future adverse chronic outcomes.

By definition,^14^ standard clinical imaging (i.e. MRI and CT) does not detect mTBI. Other neurophysiological assessments, including blood biomarkers, experimental neuroimaging sequences and analytical techniques, have proposed methods for investigating mTBI. However, none have demonstrated clinical efficacy beyond detecting intracranial bleeds.^15,16^ The mTBI pathophysiology is primarily described as a functional and microstructural injury.^17^ Animal and human research indicate that microscopic mechanical damage to axons is the most likely pathophysiological model for mTBI.^11,18,19^ White-matter (WM) connections in the brain are particularly vulnerable to impairment from biomechanical forces due to their organization and viscoelasticity. The high-velocity stretch/sheer effect caused by blast and blunt head impact contributes to axolemma mechanoporation, widespread ionic imbalances, impaired neurotransmission and metabolic disturbances that induce impaired connectivity across brain networks.^20,21^ There is a critical need to understand how mTBI exposure influences brain network connectivity. Describing ongoing, post-recovery alterations, that are at present undetectable using standard clinical imaging approaches, may contribute to future development.

Both structural and functional network neuroimaging methods have been applied to study connectivity between brain regions attributed to axonal dysfunction following mTBI. These include diffusion tensor imaging (DTI), an indirect measure of WM tract integrity and resting state functional MRI (fMRI), which measures temporal synchronization of blood-oxygenation-level-dependent (BOLD) signals between brain regions. Studies examining WM integrity using DTI in military service members have produced varying results. Standard DTI metrics across selected regions of interest (ROI) were not able to discriminate mTBI groups from post-traumatic stress disordered (PTSD) or healthy controls.^22^ However, tract-based statistics, without preselected ROIs, have indicated WM abnormalities in multiple regions.^23^ Functional neuroimaging studies have identified alterations in connectivity acutely and subacutely following mTBI.^24–26^ However, these studies analyse hyperconnectivity and hypoconnectivity between predefined regions and networks, commonly the default-mode network, using independent component analysis.^27–29^ Military TBI populations experience brain injuries from multiple exposure types—blast and/or blunt mechanisms—that may limit the effectiveness of hypothesis-driven approaches with predefined brain networks.^30,31^

Recently, the field of graph theory has proven to be a promising technique applied to fMRI, offering new ways to describe complex brain networks.^32–34^ We chose to use whole-brain graph theory rather than regional network connectivity approaches to detect global patterns in the absence of brain injury symptoms in clinically recovered soldiers. This approach may be more sensitive to detect increased risk for long-term neurological consequences. Whole-brain graph theory quantifies properties of the entire network (e.g. how nodes in the whole brain tend to be connected to other nodes with similar architecture). Traditional connectivity-based approaches detect specific regions in some participants which may demonstrate relatively stronger connectivity than other participants. Whole-brain networks consistently demonstrate ‘small world topology’, described as having high clustering and short path length, with high-degree cortical ‘hubs’ and modular and hierarchical properties.^35,36^ These features facilitate efficient local and global communication. Disruptions to this modular structure have been observed in moderate-to-severe military and civilian TBI.^31,37^ Examinations of mTBI in athletes show reduced global efficiency (GE) at both injury and 1 year post return to play in those with atypical recovery.^38^ However, there is less evidence regarding the changes to brain network topology following mTBI in military populations.^39^

Numerous metrics exist to characterize the weighted, undirected, functional brain network data obtained via fMRI. For this study, we chose GE, local efficiency (LE) and assortativity to quantify neural segregation, integration and resilience, respectively. GE is the average inverse shortest path length for all nodes. In contrast, LE measures the average GE of subgraphs for each node containing that node’s neighbours. GE quantifies the integrated exchange of information on a large scale, while LE quantifies the efficiency of segregated processes within a given node’s local neighbours.^34^ The assortativity coefficient (AC) is mathematically defined as the correlation coefficient between the degrees of all nodes on two opposite ends of a link. When quantified, AC is interpreted as the extent to which a network can resist failures in its main components.^33,34^ Networks with a greater AC have a more resilient core of interconnected, high-degree hubs. We hypothesize that a greater mTBI history would be associated with diminished brain network integration, segregation and resilience. The purpose of this study was to compare LE, GE and assortativity in SOF soldiers with and without mTBI history. The study’s second aim was to determine the dose response of multiple mTBI and mTBI recency effects on LE, GE and AC in soldiers who sustained mTBI.

## Materials and methods

### Participants

This cross-sectional study utilized data collected over 6 years (2015–21) at The University of North Carolina at Chapel Hill. This study sample included 152 healthy, asymptomatic, male, SOF combat soldiers. All participants completed verbal consent, and study procedures were approved by the Office of Human Research Ethics at our institution. All participants were asked to self-report dichotomized mTBI history (yes/no), total mTBI lifetime incidence (0, 1–2, 3+) and mTBI recency (‘past month’, ‘past year’ or ‘year+’). All participants had clinically recovered from any prior mTBI at time of visit.

### MRI acquisition

All participants were imaged at the University of North Carolina at Chapel Hill Biomedical Research Imaging Center. All MRI images were obtained on a 3T Biograph mMR or 3T MAGNETOM Prisma (Siemens, Erlangen, Germany). Whole-brain structural imaging included three-dimensional T_1_-weighted Magnetization Prepared Rapid Acquisition Gradient Echo [inversion time (TI) = 900 ms, repetition time (TR)= 1900 ms, echo time (TE) = 2.26 ms, 0.5 × 0.5 × 1 mm, FOV = 256 mm^3^, 192 slices]. Resting state fMRI was performed with eyes open staring at a fixation cross and acquired via T_2_*-weighted echo planar imaging (TR = 2300 ms, TE = 27 ms, flip angle = 90°, 44 slices, 3.5 × 3.5 × 3.5 mm voxel size) with interleaved slice acquisition. In total, 265 full-brain volumes were produced (10:16 min scan time).

### Anatomical data preprocessing

The T_1_-weighted (T_1_w) image was corrected for intensity non-uniformity with N4BiasFieldCorrection,^40^ distributed with advanced normalization tools (ANTs) 2.3.3^41^ and used as T_1_w reference throughout the workflow. The T_1_w reference was then skull stripped with a Nipype implementation of the antsBrainExtraction.sh workflow (from ANTs), using OASIS30ANTs as the target template. Brain tissue segmentation of CSF, WM and grey matter (GM) was performed on the brain-extracted T_1_w using FAST (FSL 5.0.9).^42^ Brain surfaces were reconstructed using recon-all (FreeSurfer 6.0.1),^43^ and the brain mask estimated previously was refined with a custom variation of the method to reconcile ANTs- and FreeSurfer-derived segmentations of the cortical GM of Mindboggle.^44^ Volume-based spatial normalization to one standard space (MNI152NLin2009cAsym) was performed through non-linear registration with antsRegistration (ANTs 2.3.3), using brain-extracted versions of both T_1_w reference and the T_1_w template.^45^

### Functional data preprocessing

For each BOLD run, the following preprocessing was performed. First, a reference volume and its skull-stripped version were generated using a custom methodology in fMRIPrep. Susceptibility distortion correction (SDC) was omitted. The BOLD reference was then co-registered with the T_1_w reference using bbregister (FreeSurfer 6.0.1) which implements boundary-based registration.^46^ Co-registration was configured with six degrees of freedom. Head-motion parameters with respect to the BOLD reference (transformation matrices, and six corresponding rotation and translation parameters) were estimated before any spatiotemporal filtering using mcflirt (FSL 5.0.9).^47^ The BOLD time series (including slice-timing correction when applied) were resampled onto their original, native space by applying the transformations to correct for head motion. These resampled BOLD time series will be referred to as preprocessed BOLD in original space or just preprocessed BOLD. The BOLD time series were resampled into standard space, generating a preprocessed BOLD run in MNI152NLin2009cAsym space. First, a reference volume and its skull-stripped version were generated using fMRIPrep. Several confounding time series were calculated based on the preprocessed BOLD: frame-wise displacement (FD), derivative of root mean square variance over voxels (DVARS) and three region-wise global signals. FD was computed using two formulations: Power (absolute sum of relative motions)^48^ and Jenkinson (relative root mean square displacement between affines).^47^ FD and DVARS were calculated for each functional run, both using their implementations in Nipype. The three global signals are extracted within the CSF, WM and whole-brain masks. Additionally, a set of physiological regressors were extracted to allow for component-based noise correction (CompCor).^49^ Principal components were estimated after high-pass filtering the preprocessed BOLD time series (using a discrete cosine filter with 128 s cut-off) for the two CompCor variants: temporal (tCompCor) and anatomical (aCompCor). tCompCor components were then calculated from the top 2% of variable voxels within the brain mask. For aCompCor, three probabilistic masks (CSF, WM and combined CSF + WM) were generated in anatomical space. This mask is obtained by dilating a GM mask extracted from the FreeSurfer’s aseg segmentation, and it ensures that components are not extracted from voxels containing a minimal fraction of GM. Finally, these masks are resampled into BOLD space and binarized by thresholding at 0.99 (as in the original implementation). Components are also calculated separately within the WM and CSF masks. For each CompCor decomposition, the *k* components with the largest singular values are retained, such that the retained components’ time series are sufficient to explain 50% of variance across the nuisance mask (CSF, WM, combined or temporal). The remaining components are dropped from consideration. The head-motion estimates calculated in the correction step were also placed within the corresponding confound file. The confounding time series derived from head-motion estimates and global signals were expanded with the inclusion of temporal derivatives and quadratic terms for each.^50^ Frames that exceeded a threshold of 0.5 mm FD or 1.5 standardized DVARS were annotated as motion outliers; in total, 152 out of 163 participant scans were retained as good quality. All resamplings can be performed with a single interpolation step by composing all the pertinent transformations (i.e. head-motion transform matrices, SDC when available and co-registrations to anatomical and output spaces). Gridded (volumetric) resamplings were performed using antsApplyTransforms (ANTs), configured with Lanczos interpolation to minimize the smoothing effects of other kernels.^51^ Non-gridded (surface) resamplings were performed using mri_vol2surf in FreeSurfer 6.0.1.

### Graph theoretical analyses

The BOLD signal was extracted from each participant’s postprocessed rsfMRI times series from the Big Brain 300 parcellation, a functional parcellation based on the Power atlas^52^ with improved coverage of subcortical and cerebellar regions,^53^ using a 4 mm spherical radius. Correlations between ROIs were calculated and Fisher *z*-transformed to generate subject-level correlation matrices. All negative correlations were set to zero. Weighted graph metric calculations were used to avoid thresholding the matrices at different correlation strengths or sparsity levels, maintaining a data-driven approach and avoiding multiple comparisions.^33^ The Brain Connectivity Toolbox was used to obtain whole-brain connectivity outcomes on undirected, weighted functional connectivity matrices.^34^

### Statistical analyses

Statistical analyses were performed using SAS 9.4 (SAS Institute Inc., Cary, NC, USA). Means [standard deviations (SDs)] were used to characterize continuous variables, and frequency distributions (and percentages) were used to characterize discrete variables. All outcome variables were assessed for normality by visually inspecting Q-Q plots and conducting Shapiro–Wilk tests. Brain network variables GE and LE significantly deviated from normal distribution, while AC was normally distributed. Independent two-sample *t*-tests or Wilcoxon rank sum tests were used to compare LE, GE and assortativity by binary mTBI history (yes versus no). General linear models (linear regression or Kruskal–Wallis ANOVA) were fit to compare mTBI lifetime incidence and recency. For Kruskal–Wallis ANOVA tests on non-normally distributed data, mTBI incidence was binned ordinally (0, 1–2 and 3+). Pairwise *post hoc* tests were conducted where appropriate. All statistical tests were two sided with an *a priori* α level of *P* ≤ 0.05.

## Results

**Figure 1:**
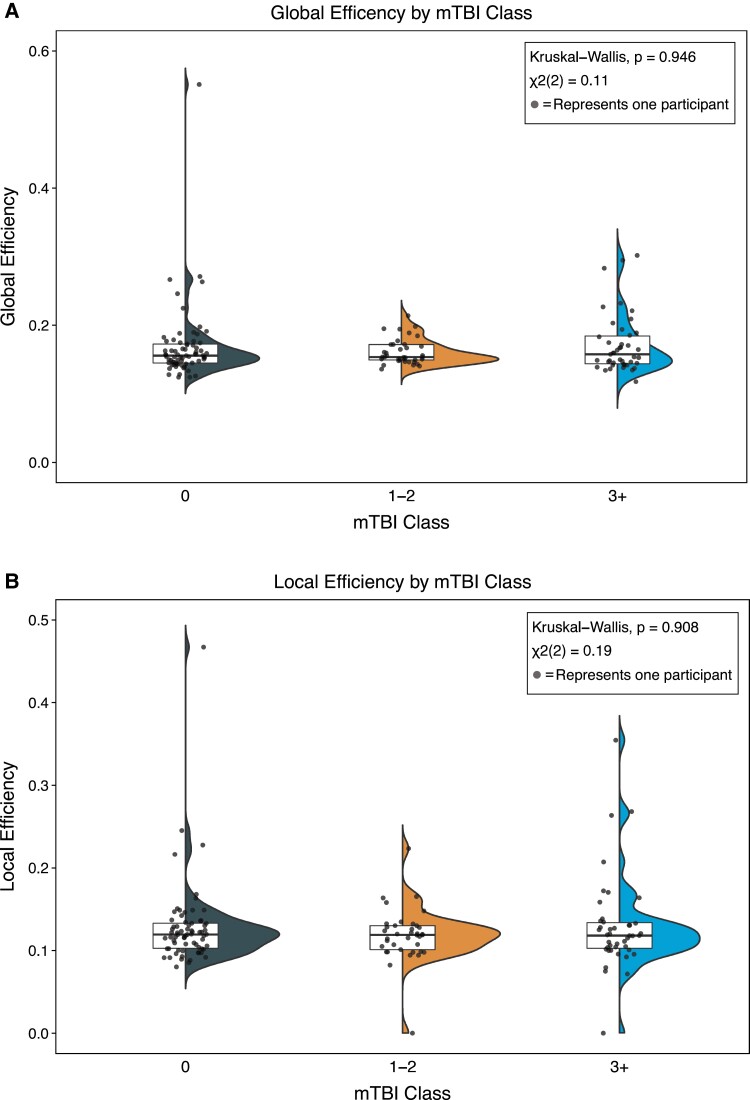
**GE (A) and LE (B) values by mTBI class are presented in this figure as half-violin plots.** These depict the non-normal distribution. The boxplot represents the median (horizontal bar). Each point reflects a single SOF combat soldier.

**Figure 2:**
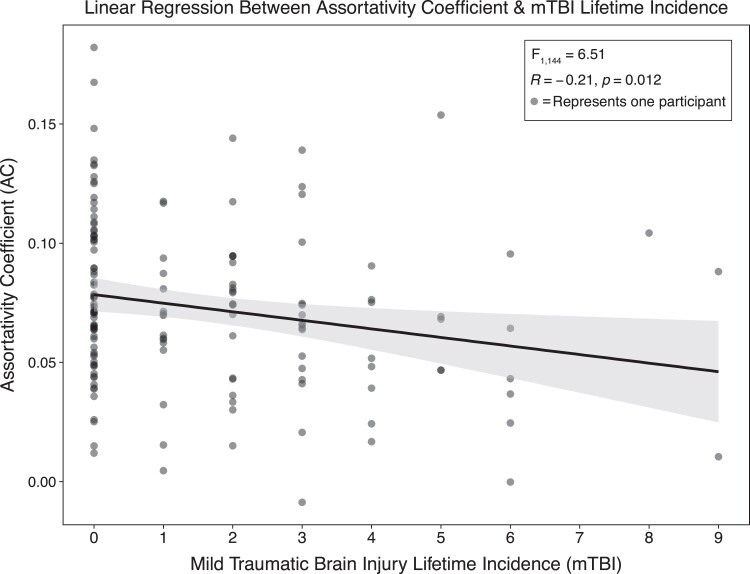
**Predicted AC by continuous mTBI lifetime incidence is depicted in this regression model.** Each point in the scatterplot represents a single SOF soldier’s AC value.

Eight participants were removed from regression models due to non-continuous mTBI data. Table 1 provides demographic data on all 152 SOF combat soldiers enrolled in our study and those included in all binary analyses. Soldiers with a history of mTBI had significantly lower AC than those who did not self-report mTBI (*t*_148_ = 2.44, *P* = 0.016). There were no significant differences in GE (*z* = −0.29, *P* = 0.775) or LE (*z* = 0.25, *P* = 0.806) for mTBI history. No differences between mTBI recency groups were observed for AC [*F*_2,68_ = 0.50, *P* = 0.611], GE [*χ*^2^(2) = 4.57, *P* = 0.102] or LE [*χ*^2^(2) = 2.29, *P* = 0.318]. We also did not observe differences between mTBI lifetime incidence groups (0, 1–2 and 3+) for GE [*χ*^2^(2) = 0.11, *P* = 0.946] or LE [*χ*^2^(2) = 0.19, *P* = 0.908; Fig. 1]. Continuous mTBI lifetime incidence significantly predicted AC [*F*_1,144_ = 6.51, *P* = 0.012], demonstrating a negative linear relationship while controlling for age via backwards elimination (*B* = −0.00359, *R*^2^ = 0.0432) with a standardized coefficient β = −0.20792 (Fig. 2).

**Table 1:** Demographic data describing the study sample size, age, mTBI history and mTBI recency for the total sample and for those who did and did not self-report mTBI history

Variable	No mTBI history	mTBI history	Total
Frequency (%)	70 (46.05%)	82 (53.95%)	152 (100%)
Mean age (SD), years	31.9 (3.7)	34.1 (4.5)	33.2 (4.3)
Median total reported mTBI (IQR)		3 (2)	1 (3)
mTBI lifetime incidence			
0	70 (100%)		70 (46.05%)
1–2		37 (45.12%)	37 (24.34%)
3+		43 (52.44%)	43 (28.29%)
Lifetime incidence not reported		2 (2.44%)	2 (1.32%)
mTBI recency^a^			
Past month		8 (9.76%)	8 (5.26%)
Past year		12 (14.63%)	12 (7.89%)
Year+		51 (62.20%)	51 (33.55%)
Recency not reported		11 (13.41%)	11 (7.24%)

^a^ Cell percentages relative to column total frequency. IQR, interquartile range; SD, standard deviation.

## Discussion

Soldiers with a greater lifetime mTBI incidence demonstrate comparatively weaker brain network resilience (i.e. low AC). Describing how soldiers recover physiologically from mTBI and detecting risk factors for short- and long-term adverse outcomes is essential for maintaining force health and readiness. It is also critically important to ensure that service members remain healthy as they transition from service into civilian retirement. Our aim was to identify neuroimaging biomarkers that reflect ongoing physiological abnormalities following mTBI recovery in SOF soldiers using whole-brain graph theoretical analyses that may link previous repetitive neurotrauma to future neurological sequelae. This study found no significant differences in global or LE between mTBI history, lifetime mTBI incidence or mTBI recency groups. However, there was a significant relationship between lower assortativity, a measure of network resilience and greater mTBI lifetime incidence. These findings suggest that efficiency, a proxy for functional capacity, may be preserved in clinically recovered soldiers following repetitive mTBI. Yet, clinically recovered patients with greater mTBI history have less neurological resilience.

**Figure 3:**
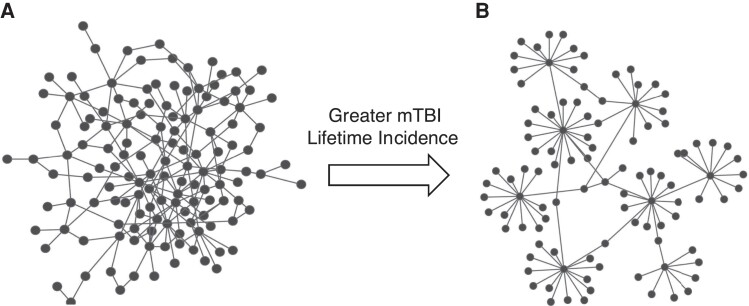
**An illustration of an assortative network (A) and a disassortative network (B).** Brain networks with greater ACs have more resilient, interconnected hubs, while a lower AC indicates widely distributed vulnerable hubs. Figure adapted from Hao and Li.^54^

The AC can also be interpreted as a network’s future tolerance for disruptions (e.g. brain injury or age-related neurodegeneration). Brain networks with greater AC have more resilient, interconnected hubs (Fig. 3A), while lower AC indicates distributed hubs that are subsequently vulnerable to prospective faults (Fig. 3B).^54^ This may explain why short-term clinical symptoms from mTBI generally resolve in patients with repetitive mTBI despite an increasing risk for neurocognitive disorders and neurological diseases. An alternative interpretation due to this study’s cross-sectional nature is that the lower assortativity observed in soldiers with greater mTBI lifetime incidence could also indicate a greater vulnerability to developing mTBI symptoms following a subsequent head impact or blast. Specifically, those with lower assortativity may have a lower injury threshold compared with those with greater assortativity. This study only examined soldiers who had recovered from mTBI; changes in global and LE metrics following mTBI may reflect the transient deficits in functional capacity that clinically resolve.

Despite our null results for efficiency metrics, mixed findings have been observed for GE and other network integration metrics in civilians and veterans with mTBI. These metrics characterize the brain’s ability to rapidly integrate information from distributed regions. Individuals with chronic TBI (8 years post injury on average) and upper moderate disability to lower good recovery on the Extended Glasgow Outcome Scale continued to show reduced global and LE compared with healthy controls.^55^ A subset of 21 civilian patients with severe-to-mild TBI had longer average path length reduced overall network efficiency compared with healthy controls as well.^56^ In 208 Operation Enduring Freedom Veterans with PTSD symptoms, LE was not associated with mTBI either; however, mTBI status moderated the association between LE in the caudate and days unable to carry out usual activity. Our findings, in context with previous studies, indicate that clinically recovered mTBI patients without persistent symptoms do not have disrupted local or GE. Therefore, lower resilience may be driving future risk for adverse long-term neurological outcomes in recovered mTBI patients.

Few studies have assessed assortativity and similar network resilience outcomes (e.g. degree distribution) following mTBI. Severe TBI patients demonstrate changes in network degree distribution, which represents the distribution of the links of all nodes in a network^33^ (i.e. an index of centrality and characterizes the resilience of the network). Specifically, severe TBI patients have more low-degree nodes and the loss of high-degree hubs which may lead to vulnerability to insult and less resilience to random gradual deterioration.^34,57,58^ In the absence of focal brain lesions, as in mTBI, the AC may be a more useful metric for future tolerance to faults in the network such as injury or neurodegenerative disease. Lower assortativity has been observed in both Alzheimer’s patients and older adults.^59,60^ Interestingly, decreasing assortativity has also been noted as an effect of chronic sleep restriction.^61^ The AC is an underexplored network metric which may offer a useful method for quantifying the cumulative neurophysiological effects of mTBI and linking them to future neurological impairment risk.

Several limitations and future directions should be acknowledged. Initially, our analysis relied on self-reported mTBI history, which introduces the risk of underreporting due to ambiguous diagnostic criteria and non-disclosure. Our design was cross-sectional in nature, and while the results are promising, no causal link can be established at this time between mTBI and decreasing assortativity. We selected a whole-brain atlas that offered good cortical and subcortical coverage across 300 ROIs. Nonetheless, there is no consensus regarding optimal atlases for network construction, and different atlases could influence network topology. Future cross-sectional studies should attempt to replicate this study in other high mTBI-risk populations such as athletes. Longitudinal prospective research is needed to assess how mTBI affects assortativity and other network measures and how these findings relate to future clinical outcomes.

## Conclusion

The present study is the first to capture LE, GE and AC in otherwise healthy soldiers with mTBI exposure. The AC, which represents a network’s resistance to damage to its main components, was lower in asymptomatic SOF soldiers with a greater mTBI lifetime incidence compared with those with lower or no mTBI history. No differences in local or GE were observed. These findings indicate that mTBI exposure may lower brain network resilience, which could increase the risk for future neurological clinical consequences.

## Data Availability

The data that support the findings of this study are available from the corresponding author upon reasonable request.
